# Genomic Signature of Oral Squamous Cell Carcinomas from Non-Smoking Non-Drinking Patients

**DOI:** 10.3390/cancers13051029

**Published:** 2021-03-01

**Authors:** Kendrick Koo, Dmitri Mouradov, Christopher M. Angel, Tim A. Iseli, David Wiesenfeld, Michael J. McCullough, Antony W. Burgess, Oliver M. Sieber

**Affiliations:** 1 Personalised Oncology Division, The Walter and Eliza Hall Institute of Medial Research, Parkville, VIC 3052, Australia; kendrick.koo@unimelb.edu.au (K.K.); mouradov.d@wehi.edu.au (D.M.); tburgess@wehi.edu.au (A.W.B.); 2 Department of Medical Biology, The University of Melbourne, Parkville, VIC 3052, Australia; 3 Department of Surgery, The Royal Melbourne Hospital, The University of Melbourne, Parkville, VIC 3050, Australia; iselient@hotmail.com (T.A.I.); david.wiesenfeld@mh.org.au (D.W.); 4 Melbourne Dental School, The University of Melbourne, Carlton, VIC 3053, Australia; m.mccullough@unimelb.edu.au; 5 Peter MacCallum Cancer Centre, Parkville, VIC 3000, Australia; christopher.angel@petermac.org; 6 Department of Biochemistry and Molecular Biology, Monash University, Clayton, VIC 3800, Australia

**Keywords:** oral cancer, tobacco, alcohol, human papilloma virus, targeted sequencing, DNA copy number, *TP53*, *CDKN2A*, *EGFR*, *PIK3CA*

## Abstract

**Simple Summary:**

A clinically distinct cohort of non-smoking non-drinking patients who develop oral cavity squamous cell carcinomas has been identified, with previous work suggesting that these patients tend to be older, female, and have poor outcomes. Our study characterised tumour molecular alterations in these patients, identifying differences in genomic profiles as compared to patients who smoke and/or drink. Associations between molecular alterations and other clinical and pathological characteristics were also explored.

**Abstract:**

Molecular alterations in 176 patients with oral squamous cell carcinomas (OSCC) were evaluated to delineate differences in non-smoking non-drinking (NSND) patients. Somatic mutations and DNA copy number variations (CNVs) in a 68-gene panel and human papilloma virus (HPV) status were interrogated using targeted next-generation sequencing. In the entire cohort, *TP53* (60%) and *CDKN2A* (24%) were most frequently mutated, and the most common CNVs were *EGFR* amplifications (9%) and deletions of *BRCA2* (5%) and *CDKN2A* (4%). Significant associations were found for *TP53* mutation and nodal disease, lymphovascular invasion and extracapsular spread, *CDKN2A* mutation or deletion with advanced tumour stage, and *EGFR* amplification with perineural invasion and extracapsular spread. *PIK3CA* mutation, *CDKN2A* deletion, and *EGFR* amplification were associated with worse survival in univariate analyses (*p* < 0.05 for all comparisons). There were 59 NSND patients who tended to be female and older than patients who smoke and/or drink, and showed enrichment of *CDKN2A* mutations, *EGFR* amplifications, and *BRCA2* deletions (*p* < 0.05 for all comparisons), with a younger subset showing higher mutation burden. HPV was detected in three OSCC patients and not associated with smoking and drinking habits. NSND OSCC exhibits distinct genomic profiles and further exploration to elucidate the molecular aetiology in these patients is warranted.

## 1. Introduction

Squamous cell carcinomas of the head and neck (HNSCC) are a heterogeneous group of cancers arising in the upper aerodigestive tract, with oral cavity cancers being the most common. HNSCC is traditionally viewed as a disease of smokers [1] and drinkers [2], but non-smoking non-drinking patients (NSND) also develop HNSCC. Chronic exposures to heavy metals from sources other than tobacco, such as contaminated food and soil, may also constitute a risk factor [3]. The human papilloma virus (HPV) is more common in oropharyngeal patients with no tobacco risk factors [4] and has a clear role in the development of oropharyngeal SCCs, but its role in oral cavity SCC (OSCC) patients without tobacco or alcohol risk factors remains poorly defined [5].

Retrospective audits of OSCC patients at our centre have revealed a larger than expected group of non-smoking (40%) and NSND (24%) patients who are predominantly female, have a bimodal age distribution, and a predilection for disease on the oral tongue. Furthermore, NSND patients with OSCC appear to have worse disease-specific mortality than smoking or drinking (SD) patients [6,7]. Other retrospective studies have also explored this NSND group, and whilst they concur that the group is more likely to be female and have oral cavity tumours, no consensus pattern in age distribution or survival outcomes has emerged [8,9,10,11,12,13,14,15]. One previous study reported poorer survival in the NSND group, but this was confined to young NSND patients [12], whilst another found a non-significant trend towards improved survival in the NSND group as a whole [11].

NSND patients are unlikely to be a homogenous group, and the suggested bimodal age distribution and adverse clinical outcomes of NSND patients highlight these patients as an important group requiring further study. Delineation of molecular alterations in NSND patients may provide insights into the aetiology of OSCC in these patients.

**Figure 1:**
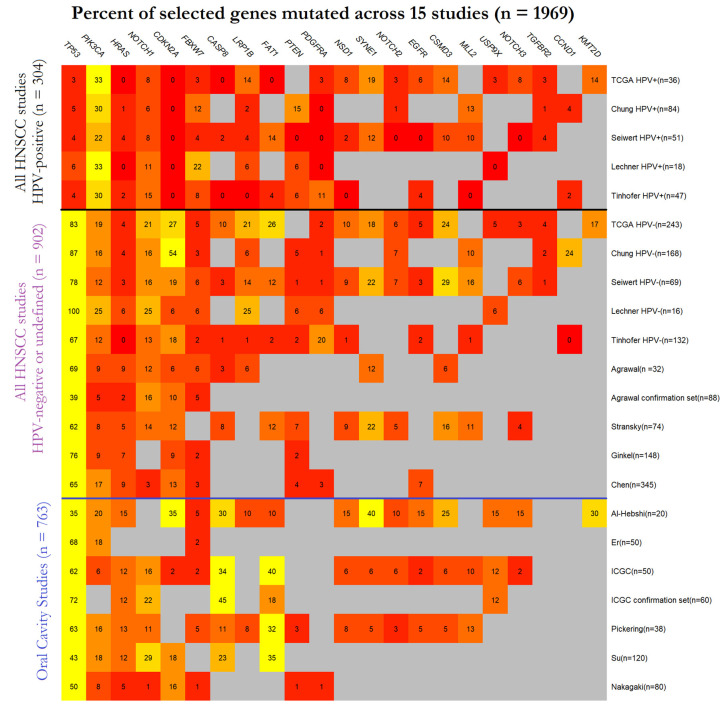
Summary of Squamous cell carcinomas of the head and neck (HNSCC) gene mutations reported in 15 previous studies [16,17,18,19,20,21,22,23,24,25,26,27,28,29,30], stratified by human papilloma virus (HPV) status as available. Studies dedicated to oral squamous cell carcinomas (OSCC) are shown separately. Percentage of patients with a gene mutation are shown; red indicates low percentages and yellow indicates high percentages. Grey boxes indicate that no data were available for that gene for a particular publication.

Recent high-throughput sequencing studies have defined the broad mutation landscape and key genomic drivers of HNSCC [16,17,18,19,20,21,22,23,24,25,26,27,28,29,30]. A few papers have specifically examined oral cavity tumours [20,22,23,24,27,29,30] but many combine HNSCC from all anatomical sites, and only a few publications separate out HPV-positive and HPV-negative tumours [16,17,19,21,28]. None of these previous papers have reported on mutations characteristic of NSND patients. A summary of principal molecular findings from previous studies of HNSCC cohorts is provided in Figure 1 [16,17,18,19,20,21,22,23,24,25,26,27,28,29,30]. Overall, these data highlight the central role of p53 inactivation in HNSCC development, with 60% of tumours (1187/1969, 60%) across studies harbouring *TP53* mutations. *CDKN2A* (315/1969, 16%), *PIK3CA* (302/1969, 15%), *NOTCH1* (230/1969, 12%) and *FAT1* (180/1969, 9%) constitute the next four most frequently mutated genes. HPV-positive tumours show distinct molecular profiles as compared to HPV-negative tumours, with less frequent mutations in *TP53* (4%, 10/236 vs. 68%, 1177/1733, *p* < 0.001), *HRAS* (2%, 4/236 vs. 7%, 110/1683, *p* < 0.01), *CASP8* (1%, 1/134 vs. 14%, 117/838, *p* < 0.001) and *CDKN2A* (0%, 0/236 vs. 20%, 315/1585, *p* < 0.001), and an enrichment of *PIK3CA* mutations (29%, 68/236 vs. 14%, 234/1673, *p* < 0.001). Comparing studies specific for OSCC to those including all head and neck sites, there is an enrichment for *CASP8* (28%, 82/288 vs. 5%, 36/684, *p* < 0.001) and *FAT1* mutations (30%, 87/288 vs. 14%, 93/652, *p* <0.001).

The impact of risk factors on somatic mutation load may also contribute to the clinical course of NSND patients: Tobacco use has been associated with a distinct somatic mutation signature in HNSCC with an enrichment of C > A transversions, although this signature appears much more pronounced in laryngeal cancers than OSCC [31]. Furthermore, a mutation signature related to APOBEC cytidine deaminase editing has been identified in HPV-positive HNSCC [32]. Notably, alcohol consumption has been associated with T > C transitions in oesophageal [33] and hepatocellular [34] carcinomas, although this has not been reported for HNSCC.

Apart from somatic mutations, HNSCCs exhibit significant genomic instability. Many HNSCCs show abundant DNA copy number variations (CNV), with prominent amplifications of chromosome 3q26/28 (the locus containing the *PIK3CA* oncogene), deletions of chromosome 9p21.3 (containing the *CDKN2A* tumour suppressor) as well as focal amplifications of *EGFR* and *CCND1*, and deletions of *FAT1* and *NOTCH1* [28]. There is one report on CNVs in a small cohort of non-smokers with oral tongue cancers that found no genomic differences as compared to smokers [35], but CNVs in the NSND group of HNSCC patients has not been addressed previously.

To refine our understanding of gene mutation profiles and somatic CNVs in OSCC and to elucidate potential genomic associations with tobacco and alcohol consumption, we performed targeted sequencing of 176 OSCCs from a community-based patient cohort for a panel of 68 frequently mutated HNSCC genes. To examine the involvement of HPV in OSCC from NSND and SD patients, our amplicon panel also included the genomes of the four most prevalent HPV risk subtypes (HPV subtypes 16, 18, 33, and 35). Mutation data were interrogated for associations with patient reported smoking and drinking habits, HPV status, clinicopathologic data, and survival outcomes.

## 2. Materials and Methods

*Patients.* A total of 176 patients with newly diagnosed OSCC presenting to the Royal Melbourne Hospital, Parkville, Australia, were examined. This study was approved by the relevant Human Research Ethics committees (RMH HREC 2013.087, RMH HREC 2012.071). For 103 patients diagnosed between January 2007 and August 2010, archival tumour blocks were retrieved from pathology archives. Regions of tumour with >50% neoplastic cell content were marked out by a specialist head and neck pathologist (C.M.A.) based on hematoxylin and eosin (H&E) stained sections, and macrodissected from 10 µm unstained serial sections. For 73 patients diagnosed between January 2014 and July 2016, fresh tumour and blood samples were obtained at surgery. Fresh-frozen tumour tissue was embedded in OCT medium and assessed for adequate (>50%) neoplastic cell content based on H&E-stained sections.

Disease stage at presentation was classified according to the AJCC 7th edition [36]. Patient smoking and drinking habits were recorded. Individuals who had smoked less than 100 cigarettes in their lifetime were classified as non-smokers, with all patients who were current or former smokers classified as smokers. Individuals without regular alcohol consumption (<1 standard drink per week) were classified as non-drinkers. All patients were treated by radical intent surgery and referred for adjuvant radiotherapy (with or without chemotherapy) as clinically appropriate. Clinical, treatment, and follow-up details were collected in a dedicated database, with a census date set at 1/1/2020 (minimum patient follow-up time of 3.5 years). Follow up was performed in line with current clinical guidelines, with disease-free patients discharged after 5 years.

*Targeted gene panel sequencing.* HNSCC somatic mutation and RNASeq data for 313 patients with oral cavity SCC were retrieved from the TCGA data portal and analysed to select genes for the curation of a dedicated 500 kb custom Agilent SureSelect XT2 amplicon panel for next-generation sequencing. Gene selection was based on mutation prevalence, RNA expression, and likelihood of contributing to oncogenesis as assessed by two previously described algorithms, OncodriveClust [37] and MutSigCV [38]. The finalised panel included 68 candidate genes, achieving a mean coverage of 95% (range 86–100%, Supplementary Table S1). To enable tumour typing for HPV status, HPV genomes for the four main high-risk subtypes (HPV subtypes 16, 18, 33, and 35) were included. DNA was extracted using the DNeasy Blood & Tissue, AllPrep DNA/RNA Mini and GeneRead FFPE extraction kits (Qiagen), according to manufacturer’s instructions. Libraries were prepared using the Agilent SureSelect XT2 system and single-end sequencing performed on an Illumina Next-Seq platform.

*Mutation detection.* Raw data were processed and mutation calling performed using GATK software [39,40]. Local realignment and base recalibration steps were performed prior to variant calling. Identified SNPs and indels were filtered and annotated with SnpEff [41]. Mutations identified exclusively on forward or reverse reads were found to be enriched in the FFPE samples as compared to the fresh-frozen samples, a known FFPE sequencing artefact [42]. Accordingly, a strand bias filter removing any mutation calls based solely on forward or reverse reads was applied across all samples to remove such sequencing artifacts.

For fresh-frozen tumour samples, somatic mutations were identified based on the sequencing data from the matched blood samples. Matched normal samples were not available for FFPE tumour samples, and putative somatic mutations were identified by filtering against germline variants identified in the 1000 Genomes Project, the normal samples from our prospective cohort and a previously curated database created for identification of somatic mutations in colorectal cancer cell lines [43]. Pathogenicity prediction was performed using the previously published PolyPhen-2 algorithm, with scores above 0.85 considered to be likely pathogenic [44].

*HPV detection.* Read counts mapping to viral sequences were normalised against library size. Samples with post-normalisation read counts for any single HPV subtype of greater than 1000 were considered to be HPV-positive.

*DNA copy number analysis.* DNA copy number analysis was conducted using ExomeDepth [45], which has been demonstrated to be a robust technique for determination of CNVs from targeted capture sequencing data [46]. A variant of the standard ExomeDepth pipeline was used [47], whereby low mappability regions as computed for 36-mers were removed from the SureSelect probe set prior to read mapping [48], with blood samples used as a reference set.

*Statistical Analysis.* All statistical analyses were performed using the *R* software for statistical computing [49]. Differences between groups were assessed using Fisher’s exact test for categorical variables and the Kruskal Wallis test for continuous variables. Mutation counts were compared between groups of interest using a generalised linear model [50]. Each gene mutated in at least 5% of patients (mutations in >10 cases) and with at least 50% of mutations assigned as likely pathogenic were correlated to clinicopathologic variables. Between-group survival differences by mutation status were assessed using Kaplan–Meier analysis and Cox-proportional hazard models adjusting for clinicopathologic variables. Overall survival was defined as time from diagnosis to death, with censoring done where patients were alive at last contact. Two-sided *p*-values < 0.05 were considered statistically significant.

## 3. Results

### 3.1. Patient Clinical Characteristics and HPV Status

Clinical details of 176 OSCC patients examined in this study are summarised in Table 1. A total of 82 patients had early stage (stage I/II) disease and 94 patients had local or regionally advanced disease (stage III/IV). All patients were treated with radical intent surgery and were referred for radiotherapy and/or chemotherapy following discussion at a multidisciplinary team meeting. Sixty-three percent (110/176) of patients received adjuvant radiotherapy and 22% (39/176) were treated with chemotherapy.

Clinicopathologic details and treatment delivery were similar between retrospective patients (n = 103) diagnosed between January 2007 and August 2010 and prospectively recruited patients (n = 73) diagnosed between January 2014 and July 2016. However, the proportions of non-drinkers and NSND patients were higher in the prospective cohort, consistent with the reported trend of reduced alcohol consumption among Australians over this time period [51] (Supplementary Table S2).

**Figure 2:**
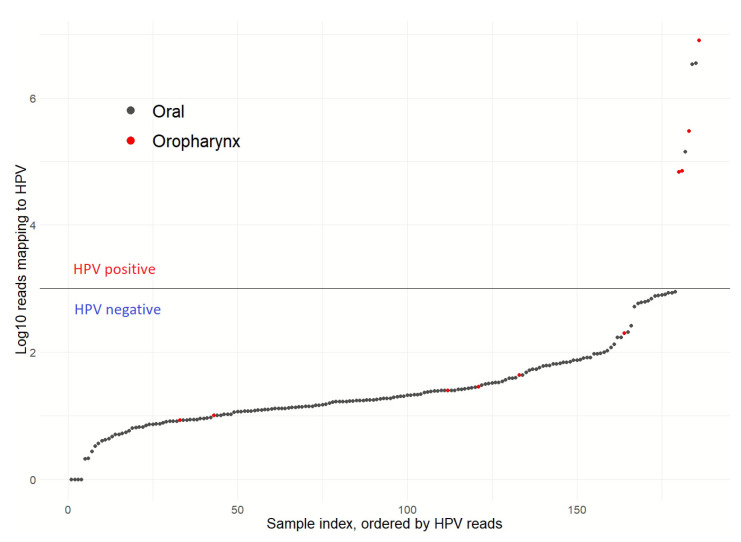
HPV prevalence in 176 OSCC patients for high-risk HPV subtypes 16, 18, 33, and 35 based on genomic sequencing. Tumour samples with normalised HPV read counts >1000 were considered HPV-positive. Seven oropharyngeal tumours, which are known to have a high prevalence of HPV infection, were included as control.

Presence of HPV was identified through our targeted sequencing approach in 3 out of 176 (1.7%) OSCCs **(**Figure 2); one case was positive for HPV-16 and two cases for HPV-33. This HPV detection rate is consistent with a previous study from our centre, which used orthogonal methods (PCR-ELISA and RNA in situ hybridization) to identify HPV [52] and all of the overlapping patients between the two studies had concordant HPV detection results (39/39 patients, 2/39 HPV-positive), supporting accuracy of targeted next generation sequencing for virus detection. As a further control, a small set of prospectively collected oropharyngeal tumours, which are known to have high prevalence of HPV infection [5], were also sequenced with 57% (4 out of 7) tumours found to be positive for HPV-16, consistent with the prevalence reported by a previous systematic review [53]. A single OSCC NSND patient (1.7%, 1/59) was HPV-positive, similar to the HPV-positive rate in SD patients (1.7%, 2/117, *p* = 1). There were no significant associations between HPV status and clinicopathologic variables in OSCC patients (Supplementary Table S3).

**Figure 3:**
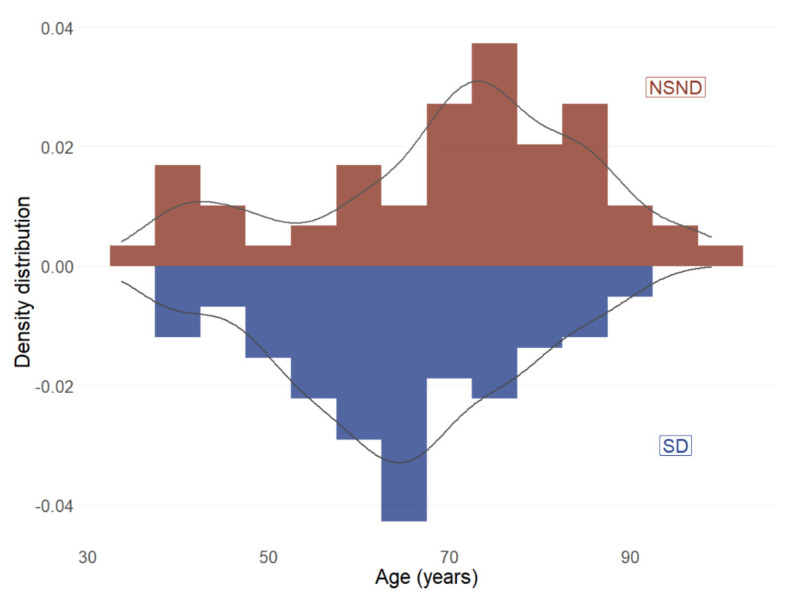
Age of diagnosis distribution for 176 OSCC patients by drinking and smoking status. NSND = non-smoker and non-drinker; SD = smokers and/or drinker.

NSND patients were significantly older than SD patients (mean age of 70 years vs. 64 years, *p* = 0.004). However, there was evidence for a bimodal age distribution (Figure 3), consistent with our previously reported findings that included a subset of the current cohort [6]. As anticipated, a significantly higher proportion of NSND patients (73%, 43/59) were female as compared to SD patients (28%, 28/117; *p* < 0.001), while other clinical features were similar (Supplementary Table S4). NSND patients showed poorer five-year overall survival as compared to SD patients in univariate analysis (HR 1.7, 95% CI 1.0–2.8, *p* = 0.05, Supplementary Figure S1), although this was not maintained in multivariate analysis adjusting for clinicopathologic features (Supplementary Table S5).

### 3.2. Genomic Alterations and Clinical Associations for OSCC Patients

Non-synonymous somatic mutations in 68 cancer genes were identified in 93% (164/176) of OSCC patients (Supplementary Data) with similar mutation frequencies in tumours from prospective and retrospective patients (*p* = 0.25 by Kruskal-Wallis).

**Figure 4:**
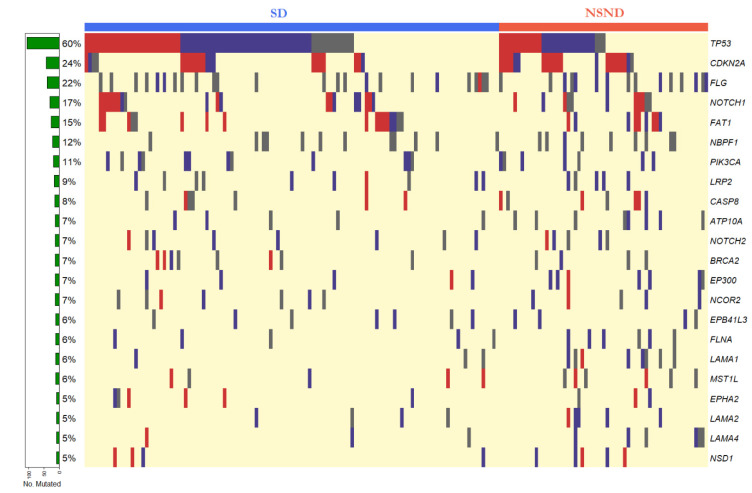
Mutation map for 23 candidate genes mutated in at least 5% (9/176) of tumours from OSCC patients. Nonsense and indel mutations are indicated by red bars, missense mutations with a PolyPhen-2 score > 0.85 are indicated by purple bars, missense mutations with a PolyPhen-2 score < 0.85 indicated by grey bars. The row at the bottom indicates patients with no detected mutations in the targeted sequencing panel. The colour bar at the top denotes smokers and/or drinkers (SD, blue) and non-smokers and non-drinkers (NSND, red).

Seven genes had mutations in greater than 10% of samples, including *TP53* (60%, 106/176), *CDKN2A* (24%, 42/176), *FLG* (22%, 39/176), *NOTCH1* (17%, 30/176), *FAT1* (15%, 26/176), *NBPF1*(12%, 21/176), and *PIK3CA* (11%, 21/176) (Figure 4). Frequently mutated sites in key driver genes *TP53*, *CDKN2A*, and *PIK3CA* corresponded to hotspots identified by the Catalogue of Somatic Mutations in Cancer (COSMIC) database (Supplementary Figures S2–S4).

Based on the predicted pathogenicity score from the PolyPhen-2 algorithm or nonsense/indel mutation status, the majority of mutations in *TP53* (85%, 91/106), *CDKN2A* (93%, 39/42), *NOTCH1* (83%, 25/30), *FAT1* (85%, 22/26), *PIK3CA* (62%, 13/21) were likely pathogenic. In contrast, smaller proportions of mutations were assigned as likely pathogenic for *FLG* (31%, 12/39) and *NBPF1* (5%, 1/21). Additionally, likely pathogenicity was assigned for the majority of mutations in 12 out of 16 genes that exhibited mutation frequencies between 5% and 10%. These genes included *CASP8* (57%, 8/14), *NOTCH2* (69%, 9/13), *EP300* (92%, 11/12), *NCOR2* (58%, 7/12), *EPHA2* (78%, 7/9), and *LAMA2* (78%, 7/9) (Supplementary Table S6). Low levels (<5%) of mutations were found in 45 genes with no mutations detected in 4 of our candidate genes.

DNA copy-number aberrations of one or more candidate genes were identified in 64% (113/176) of tumours (Supplementary Data), with fewer CNVs detected for patients in the retrospective cohort (mean 1.0, range 0–5) as compared to the prospective cohort (mean 2.0, range 0–7, *p* < 0.01), potentially related to differential algorithm sensitivity in archival versus fresh-frozen specimens. Out of CNVs identified at similar frequencies in both groups of patients, the most frequent amplifications were detected in *EGFR* (9%, 16/176), *MMP12* (6%, 10/176) and *PRKDC* (5%, 8/176), while the most frequent deletions were in *BRCA2* (5%, 9/176 patients) and *CDKN2A* (4%, 7/176) (Supplementary Table S7). Read ratios for representative samples with *EGFR* amplification and *CDKN2A* deletion are shown in Supplementary Figures S5 and S6. No deletions characteristic of EGFRvIII were identified.

A total of 17 genes were mutated in at least 5% of patients and had at least 50% of mutations assigned likely pathogenic. Associations with clinicopathologic variables were examined for these genes as well as the five genes with recurrent CNVs (Table 2).

*TP53* mutations were significantly associated with male gender (Male: 67/100 vs. Female: 39/76, *p* = 0.043), nodal disease (N+: 48/61 vs. N0: 58/115, *p* < 0.001), lymphovascular invasion (LVI+: 15/18 vs. LVI-: 91/158, *p* = 0.042) and extracapsular spread (ECS+: 19/20 vs. ECS-: 87/156, *p* < 0.001). *CDKN2A* mutations were more frequent in non-smokers (Non-smokers: 27/86 vs. Smokers: 15/90, *p* = 0.033) and NSND patients (NSND: 21/59 vs. SD: 21/117, *p* = 0.014) and associated with advanced tumour stage (T3/4: 26/71 vs. T1/2: 16/105, *p* = 0.002) and extracapsular spread (ECS+: 9/20 vs. ECS-: 33/156, *p* = 0.026). CASP8 mutations were associated with female gender (Male: 3/100 vs. Female: 11/76, *p* = 0.009) and non-drinking status (Non-drinkers: 10/79 vs. Drinkers: 4/97, *p* = 0.0497). No associations with gender, drinking status, smoking status, tumour stage, nodal involvement, LVI, ECS and HPV status were observed for *FAT1* or *PIK3CA* mutated tumours. No HPV-positive patient (0/3) had a *TP53* mutation, but this did not reach statistical significance. *EGFR* amplification was associated with NSND status (NSND: 10/59 vs. SD: 6/117, *p* = 0.023), perineural invasion (PNI+: 7/32 vs. PNI-: 9/144, *p* = 0.012) and extracapsular spread (ECS+: 5/20 vs. ECS-: 11/156, *p* = 0.022). Copy number loss of *CDKN2A* was associated with advanced tumour stage (T3/4: 7/71 vs. T1/2: 0/105, *p* = 0.001) and loss of *BRCA2* was associated with advanced tumour stage (T3/4: 8/71 vs. T1/2: 1/105, *p* = 0.003), nodal disease (N+: 7/61 vs. N0: 2/115, *p* = 0.009), extracapsular spread (ECS+: 4/20 vs. ECS-: 5/156, *p* = 0.011) and NSND status (NSND: 7/59 vs. SD: 2/117, *p* = 0.007).

**Figure 5:**
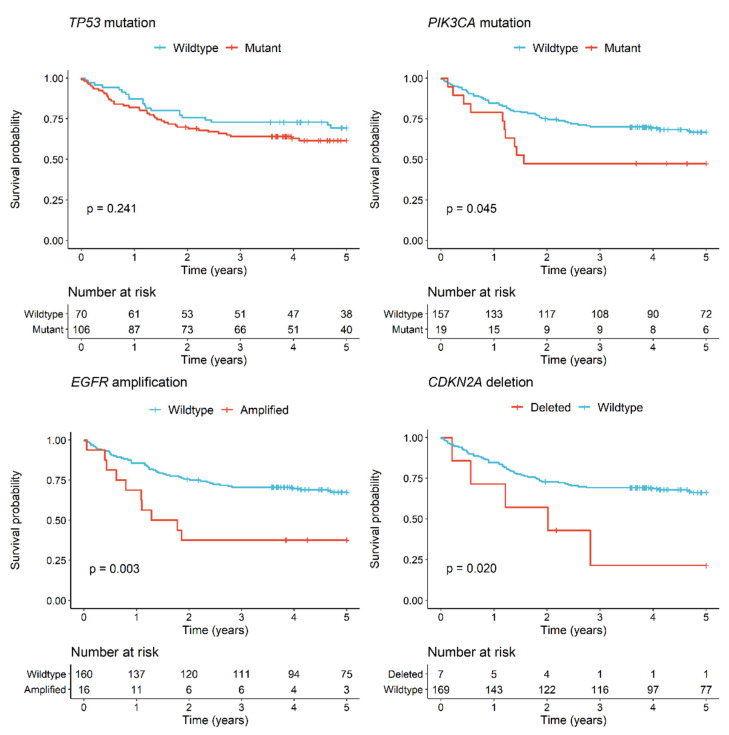
Kaplan–Meier survival curves for OSCC patients by *TP53* mutation, *PIK3CA* mutation, *EGFR* amplification, or *CDKN2A* deletion status. *p* values are for the log rank test.

Univariate analysis for five-year overall survival was not significant for *TP53* (Figure 5), *CDKN2A*, and *FAT1* (Supplementary Figure S7) mutations (*p* > 0.05). Significantly poorer outcomes were observed for patients with *PIK3CA* mutated tumours as compared to patients with *PIK3CA* wild-type tumours (HR 2.0, 95% CI 1.0–3.9, *p* = 0.045) (Figure 5) although this did not remain significant in a multivariate analysis adjusting for clinicopathologic variables (Table 3). No other gene mutation was associated with a statistically significant survival difference (Supplementary Table S8). *EGFR* amplification was significantly associated with poorer survival (HR 2.7, CI 1.4–5.4, *p* = 0.004) as was *CDKN2A* deletion (HR 2.8, CI 1.1–7.1, *p* = 0.026) in univariate analyses (Figure 5), but this was not maintained when adjusting for other variables (Table 3).

### 3.3. Mutation Differences between NSND and SD Patients

**Figure 6:**
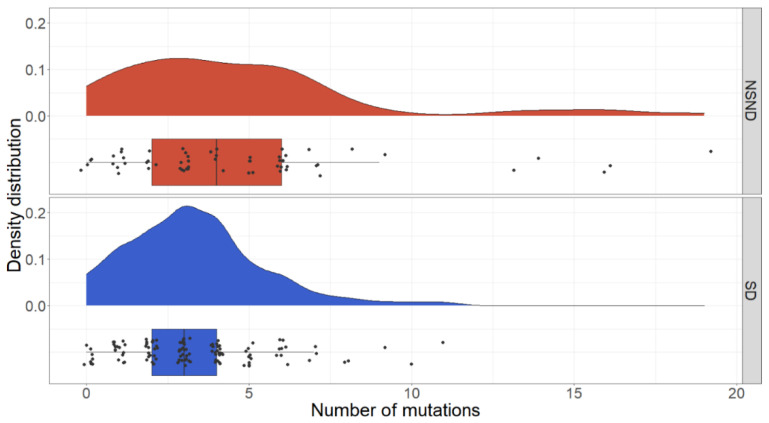
Distribution of mutation counts, comparing the NSND and the SD groups. NSND = non-smoker and non-drinker; SD = smokers and/or drinker.

We observed more mutated genes in non-drinkers (mean 4.3 vs. 3.4 in drinkers, *p* = 0.001), non-smokers (mean 4.2 vs. 3.4 in smokers, *p* = 0.008), and the NSND patients (mean 4.7 vs. mean 3.3 in SD patients, *p* < 0.001). The mutation spectrum comparing NSND to SD patients is visualised in Supplementary Figure S8. Examination of mutation counts identified five patients among the NSND group who had higher numbers of mutations (>12) as compared to the SD group (Figure 6).

These five patients were younger than the remainder of the NSND group (mean 53 years vs. 71 years, *p* = 0.013). The distribution of mutation types (transitions, transversions, and indels) in these five patients were compared to the distribution in other NSND patients as well as the SD group (Table 4). There was no significant difference between this high mutation group and the remainder of the NSND group (*p* = 0.297). However, compared to the SD group, there was a decrease in proportion of insertions/deletions, and an enrichment of T > C transitions (*p* = 0.019 for the NSND high mutation group, *p* = 0.067 for the NSND group as a whole). There was no evidence of enrichment of tobacco-associated enrichment of C > A transversions or alcohol-associated enrichment of T > C transitions among SD patients.

## 4. Discussion

This study surveyed the molecular profiles of 176 OSCC patients, 34% of which were NSND patients, providing insights into the aetiology of this subgroup. HPV was excluded as a major contributor to carcinogenesis in oral cavity cancers in the NSND group, with a similar low prevalence in both this subgroup (1.7%) and SD patients (1.7%). Nonetheless, none of the HPV-positive OSCCs in this study harboured a *TP53* mutations, consistent with the well-established role of HPV E6 protein as an inhibitor of *TP53* [54].

In the context of the targeted gene panel, a subset of our NSND OSCC patients had a higher mutation burden than SD patients. This was an unexpected finding as the *a priori* expectation was that smokers/drinkers would accumulate more mutations over time as a result of carcinogen exposure. The increase in mutation burden, particularly of T > C transitions, in the NSND group could imply an underlying mutational process, but with our limited targeted sequencing, mutational signatures could not be explored in depth. An alternate hypothesis is that the oncogenes and tumour suppressor genes targeted by our sequencing panel may play a more dominant role in NSND patients. Sequencing of the entire exome or genome and replication in an independent cohort would be required to differentiate between these possibilities.

In NSND patients, the well described tumour suppressor *CDKN2A* was found to be mutated at almost twice the frequency of SD patients (35.6% vs. 17.9%), and this was also evident when comparing smokers to non-smokers. However, the frequency of *CDKN2A* deletions was not significantly different between groups (NSND: 1/59, 1.7%; SD 6/117, 5.1%). Notably, *CDKN2A* promoter methylation is another mechanism of *CDKN2A* inactivation, which is known to be common in HNSCC as a whole (20% of cases in TCGA data [28]) but could not be evaluated in our cohort. Whilst an association between smoking and *CDKN2A* inactivation has not previously been identified in OSCC, a meta-analysis in non-small cell lung carcinoma (NSCLC) has reported a positive association between p16 promoter methylation and smoking [55].

Amplification of *EGFR* was more common in the NSND group than the SD group (16.9% vs. 5.1%). Overexpression of EGFR has been found to be correlated with smoking and poorer overall survival in oropharyngeal SCC [56], and in NSCLC, *EGFR* mutations are more common in non-smokers than smokers and is clinically helpful in guiding the use of targeted therapy [57]. In a similar vein, exploration of *EGFR* as a biomarker for EGFR-directed therapy in NSND OSCC patients may be warranted. *BRCA2* deletions were more frequently identified in the NSND group than the SD group (11.9% vs. 1.7%) although the significance of these deletions is uncertain.

Our study also highlighted a number of more general molecular associations in OSCC. *TP53* mutation was associated with nodal disease, lymphovascular invasion, and extracapsular spread, consistent with previous reports in the OSCC literature [58]. Mutations and deletions of *CDKN2A* were independently associated with advanced tumour stage in our cohort and some investigators have associated *CDKN2A* copy number loss with poor prognosis in HNSCC [59], which was also observed in univariate analysis in our patients. Finally, *EGFR* amplification was associated with poor overall survival in univariate analysis and was associated with perineural invasion and extracapsular spread. Extracapsular spread has previously been associated with *EGFR* amplification [60] or high expression levels of EGFR [61,62], as has perineural invasion [63]. Whilst overexpression of EGFR has been associated with worse survival in oropharyngeal cancers [56], previous work has not identified an association between *EGFR* amplification and survival [64]. Finally, *PIK3CA* mutations were found to be associated with poor prognosis in OSCC patients in univariate analysis, which has previously been reported in a cohort of HPV-positive oropharyngeal SCCs [65].

Caveats of our study are that tobacco and alcohol histories were self-reported and exposure to second-hand tobacco is difficult to quantify, which may lead to some erroneous classifications of NSND status. The cohort size in our study was limited although molecular findings were broadly consistent with the OSCC literature. Our survey of molecular alterations was limited to a panel of genes, precluding more detailed examination of mutation signatures or larger-scale DNA copy-number or structural alterations that may drive oncogenesis in the NSND group. In addition, transcriptomic and epigenomic alterations may contribute to OSCC in NSND patients. Examination of independent cohorts will be required to validate our findings. As the proportion of NSND HNSCC patients is relatively small, this will likely require aggregation of clinically annotated HNSCC sequencing datasets across multiple institutions.

## 5. Conclusions

In summary, we have excluded HPV as a primary driver underlying oral carcinogenesis in NSND patients and have identified significant molecular differences between the NSND and SD groups in OSCC including cancer gene alterations and mutation burden based on our targeted gene panel. Further studies are warranted to elucidate the molecular aetiology of OSCC in NSND patients.

## Figures and Tables

**Table 1:** Clinical characteristics of 176 OSCC patients in this study. Percentages for groups are shown in brackets. NSND = non-smoker and non-drinker.

Characteristic		All Patients (n = 176)
Gender	Female	76 (43.2)
	Male	100 (56.8)
Age	Median (range)	66 (33–98)
	Non-smoker	86 (48.9)
	Non-drinker	79 (44.9)
	NSND	59 (33.5)
T stage	1	39 (22.2)
	2	66 (37.5)
	3	14 (8.0)
	4	57 (32.4)
N stage	N0	115 (65.3)
	N+	61 (34.7)
AJCC stage	I	32 (18.2)
	II	50 (28.4)
	III	37 (21.0)
	IV	57 (32.4)
Perineural invasion	Present	32 (18.2)
	Absent	144 (81.8)
Lymphovascular invasion	Present	18 (10.2)
	Absent	158 (89.8)
Extracapsular spread	Present	20 (11.4)
	Absent	156 (88.6)
HPV status	Positive	3 (1.7)
	Negative	173 (98.3)
Radiotherapy	Yes	110 (62.5)
	No	66 (37.5)
Chemotherapy	Yes	39 (22.2)
	No	137 (77.8)

**Table 2:** Univariate analysis for selected gene mutations and copy number alterations against clinicopathologic variables. “Group 1” indicates the referent variable, whilst “Group 2” indicates the comparison variable. Only comparisons where *p* < 0.05 are shown. NSND = non-smoker and non-drinker; SD = smokers and/or drinker; LN = lymph node; LVI = lymphovascular invasion; PNI = perineural invasion; ECS = extracapsular spread; OR = odds ratio, CI = confidence interval; * *p* < 0.05.

Group 1 vs. Group 2	Gene	Group 1 n (%)	Group 2 n (%)	OR (95% CI)	*p*
Male vs. Female	*TP53* mut	67/100 (67.0)	39/76 (51.3)	1.9 (1.0–3.7)	0.043 *
	*CASP8* mut	3/100 (3.0)	11/76 (14.5)	0.2 (0.0–0.7)	0.009 *
Smokers vs. Non-smokers	*CDKN2A* mut	15/90 (16.7)	27/86 (31.4)	0.4 (0.2–0.9)	0.033 *
Drinkers vs. Non-drinkers	*CASP8* mut	4/97 (4.1)	10/79 (12.7)	0.3 (0.1–1.1)	0.050 *
	*LAMA4* mut	1/97 (1.0)	8/79 (10.1)	0.1 (0.0–0.7)	0.012 *
NSND vs. SD	*CDKN2A* mut	21/59 (35.6)	21/117 (17.9)	2.5 (1.2–5.5)	0.014 *
	*EGFR* amp	10/59 (16.9)	6/117 (5.1)	3.7 (1.2–13.3)	0.023 *
	*BRCA2* del	7/59 (11.9)	2/117 (1.7)	7.6 (1.4–77.8)	0.007 *
T3/4 tumours vs. T1/2 tumours	*CDKN2A* mut	26/71 (36.6)	16/105 (15.2)	3.2 (1.5–7.1)	0.002 *
	*CDKN2A* del	7/71 (9.9)	0/105 (0)	Inf (2.3–Inf)	0.001 *
	*BRCA2* del	8/71 (11.3)	1/105 (1.0)	13 (1.7–590.0)	0.003 *
LN+ vs. LN−	*TP53* mut	48/61 (78.7)	58/115 (50.4)	3.6 (1.7–8.1)	<0.001 *
	*BRCA2* del	7/61 (11.5)	2/115 (1.7)	7.2 (1.3–73.5)	0.009 *
LVI+ vs. LVI−	*TP53* mut	15/18 (83.3)	91/158 (57.6)	3.7 (1.0–20.0)	0.042 *
	*NCOR2* mut	4/18 (22.2)	8/156 (5.1)	5.3 (1.0–22.9)	0.023 *
PNI+ vs. PNI−	*EGFR* amp	7/32 (21.9)	9/144 (6.2)	4.2 (1.2–13.9)	0.012 *
ECS+ vs. ECS−	*CDKN2A* mut	9/20 (45.0)	33/156 (21.2)	3 (1.0–8.8)	0.026 *
	*TP53* mut	19/20 (95.0)	87/156 (55.8)	15 (2.3–633.0)	<0.001 *
	*EGFR* amp	5/20 (25.0)	11/156 (7.1)	4.3 (1.0–16.0)	0.022 *
	*BRCA2* del	4/20 (20.0)	5/156 (3.2)	7.4 (1.3–38.4)	0.011 *

**Table 3:** Univariate and multivariate Cox proportional hazards analysis assessing *PIK3CA* mutation, *EGFR* amplification or *CDKN2A* mutation and clinicopathologic variables in OSCC patients. NSND = non-smoker and non-drinker; SD = smokers and/or drinker; LN = lymph node; PNI = perineural invasion; LVI = lymphovascular invasion; HR = hazard ratio, AHR = adjusted hazard ratio, CI = confidence interval; * *p* < 0.05.

	Univariate Analysis			Multivariate Analysis		
	HR	95% CI	*p*	AHR	95% CI	*p*
***PIK3CA* Mutation**	2.0	1.0–3.9	0.050 *	1.4	0.7–2.9	0.303
Male vs. female	0.8	0.5–1.3	0.406	1.1	0.6–1.9	0.808
Age (in decades)	1.7	1.2–1.8	<0.001 *	1.6	1.3–2.0	<0.001 *
NSND vs. SD	1.7	1.0–2.8	0.050 *	1.2	0.6–2.1	0.630
T3/4 vs. T1/2	2.9	1.7–5.0	<0.001 *	2.6	1.5–4.4	0.001 *
LN+ vs. LN-	2.3	1.4–3.8	0.001 *	2.0	1.1–3.6	0.019 *
PNI+ vs. PNI-	1.7	1.0–3.1	0.064	1.5	0.8–2.7	0.211
LVI+ vs. LVI-	2.0	1.0–4.0	0.064	1.4	0.6–3.1	0.443
***EGFR* Amplification**	2.7	1.4–5.4	0.004 *	1.8	0.9–3.6	0.118
Male vs. female	0.8	0.5–1.3	0.406	1.1	0.6–1.9	0.861
Age (in decades)	1.7	1.2–1.8	<0.001 *	1.6	1.3–2.1	<0.001 *
NSND vs. SD	1.7	1.0–2.8	0.050 *	1.1	0.6–2.0	0.829
T3/4 vs. T1/2	2.9	1.7–5.0	<0.001 *	2.4	1.4–4.2	0.001 *
LN+ vs. LN-	2.3	1.4–3.8	0.001 *	2.0	1.1–3.7	0.016 *
PNI+ vs. PNI-	1.7	1.0–3.1	0.064	1.4	0.7–2.6	0.301
LVI+ vs. LVI-	2.0	1.0–4.0	0.064	1.5	0.6–3.3	0.360
***CDKN2A* Deletion**	2.8	1.1–7.1	0.026 *	1.8	0.6–5.0	0.261
Male vs. female	0.8	0.5–1.3	0.406	1.0	0.6–1.9	0.932
Age (in decades)	1.7	1.2–1.8	<0.001 *	1.6	1.3–2.0	<0.001 *
NSND vs. SD	1.7	1.0–2.8	0.050 *	1.2	0.6–2.3	0.556
T3/4 vs. T1/2	2.9	1.7–5.0	<0.001 *	2.3	1.3–4.1	0.004 *
LN+ vs. LN-	2.3	1.4–3.8	0.001 *	2.2	1.2–4.1	0.009 *
PNI+ vs. PNI-	1.7	1.0–3.1	0.064	1.5	0.8–2.8	0.172
LVI+ vs. LVI-	2.0	1.0–4.0	0.064	1.2	0.5–2.8	0.654

**Table 4:** Distribution of mutational alterations, comparing the SD group with the entire NSND group or subset of with low or high mutation load. NSND = non-smoker and non-drinker; SD = smokers and/or drinker. * *p* < 0.05.

Alteration	SD (n = 117, 434 Mutations)	NSND, All (n = 59, 366 Mutations)	NSND, Low Mutation Group (n = 54, 233 Mutations)	NSND, High Mutation Group (n = 5, 133 Mutations)
C > A	60 (13.8)	39 (10.7)	27 (11.6)	12 (9.6)
C>G	44 (10.1)	42 (11.5)	27 (11.6)	15 (12.0)
C>T	178 (41.0)	164 (44.8)	107 (45.9)	57 (45.6)
T>A	33 (7.6)	12 (3.3)	6 (2.6)	6 (4.8)
T > C	47 (10.8)	61 (16.7)	35 (15.0)	26 (20.8)
T>G	18 (4.1)	16 (4.4)	7 (3.0)	9 (7.2)
Indel	54 (12.4)	32 (8.7)	24 (10.3)	8 (6.0)
Compared to SD		*p* = 0.010 *	*p* = 0.067	*p* = 0.019 *
Compared to NSND				*p* = 0.297

## Data Availability

The molecular data presented in this study are available in the Supplementary Data. Associated clinical data cannot be provided to maintain patient confidentiality.

## Supplementary Materials

The following are available online at https://www.mdpi.com/2072-6694/13/5/1029/s1: Supplementary Table S1. Coverage statistics for the Agilent® SureSelect XT2 amplicon panel for 68 selected candidate HNSCC genes, Supplementary Table S2. Clinical characteristics of 73 prospective and 103 retrospective OSCC patients, Supplementary Table S3. Clinical characteristics of HPV-negative OSCC patients compared against HPV-positive patients, Supplementary Table S4. Clinical characteristics of OSCC patients in the NSND group compared to the SD group, Supplementary Table S5. Univariate and multivariate and Cox proportional hazards analyses assessing smoking/drinking status and clinicopathologic variables in 176 OSCC patients, Supplementary Table S6. Pathogenicity predictions by the PolyPhen-2 algorithm for missense mutations detected in tumours from 176 OSCC patients, Supplementary Table S7. Gene amplifications and deletions in 176 OSCC patients as determined by ExomeDepth, Supplementary Table S8. Univariate and multivariate and Cox proportional hazards analyses assessing genes mutated in at least 5% of OSCC patients, Supplementary Figure S1. Kaplan-Meier survival curves for 176 OSCC patients by smoking/drinking status, Supplementary Figure S2. Amino acid positions for TP53 mutations detected in our OSCC cohort against mutations reported for aerodigestive tumours in the COSMIC database, Supplementary Figure S3. Amino acid positions for CDKN2A mutations detected in our OSCC cohort against mutations reported for aerodigestive tumours in the COSMIC database, Supplementary Figure S4. Amino acid positions for PIK3CA mutations detected in our OSCC cohort against mutations reported for aerodigestive tumours in the COSMIC database, Supplementary Figure S5. ExomeDepth CNV plot for a representative OSCC sample with a detected EGFR amplification, Supplementary Figure S6. ExomeDepth CNV plot for a representative OSCC sample with a detected CDKN2A deletion, Supplementary Figure S7. Kaplan-Meier survival curves for 176 OSCC patients by CDKN2A, FAT1, NOTCH1 or CASP8 mutation status, Supplementary Figure S8. Spectrum of missense mutations in OSCC tumours from NSND patients as compared to SD patients: Supplementary_Figures_Tables.docx. Supplementary Data. Somatic mutations and DNA copy number variants detected in 176 OSCC patients: Supplementary_Data.xlsx.
