# Supplementary material for: Genomic Signature of Oral Squamous Cell Carcinomas from Non-Smoking Non-Drinking Patients
Source: Cancers (Basel). 2021 Mar 1;13(5):1029. doi: 10.3390/cancers13051029 (PMC7957667; doi:10.3390/cancers13051029)
Supplement: Supplementary file 1 [file cancers-13-01029-s001.zip › cancers-1113763 Supplementary_Figures_Tables .docx]

*Supplementary Materials*

**Genomic Signature of Oral Squamous Cell Carcinomas from Non-Smoking Non-Drinking Patients**

**Kendrick Koo ^1,2,3,4^, Dmitri Mouradov ^1,2^, Christopher M. Angel ^5^, Tim A. Iseli ^3^, David Wiesenfeld ^3,4^, Michael J McCullough ^4^, Antony W Burgess ^1,2,3^ and Oliver M Sieber ^1,2,3,6,^***

**Supplementary Table S1.** Coverage statistics for the Agilent^®^ SureSelect XT2 amplicon panel for 68 selected candidate HNSCC genes.

| Target ID | Regions | Coverage | High Coverage  (> = 90%) | Low coverage  ( < 90%) |
| --- | --- | --- | --- | --- |
| *AJUBA* | 8 | 100.0% | 8 | 0 |
| *APC* | 19 | 99.5% | 18 | 1 |
| *ATP10A* | 22 | 100.0% | 22 | 0 |
| *ATP13A4* | 30 | 100.0% | 30 | 0 |
| *ATR* | 49 | 99.1% | 48 | 1 |
| *BRCA2* | 26 | 100.0% | 26 | 0 |
| *CACNA1C* | 53 | 100.0% | 53 | 0 |
| *CACNA1D* | 55 | 100.0% | 55 | 0 |
| *CACNA1E* | 51 | 100.0% | 51 | 0 |
| *CACNA2D1* | 42 | 100.0% | 42 | 0 |
| *CASP8* | 13 | 95.2% | 12 | 1 |
| *CD163* | 16 | 100.0% | 16 | 0 |
| *CDKN2A* | 5 | 100.0% | 5 | 0 |
| *CHEK2* | 20 | 86.2% | 16 | 4 |
| *CREBBP* | 31 | 99.0% | 30 | 1 |
| *CTCF* | 10 | 100.0% | 10 | 0 |
| *CTNNA2* | 22 | 100.0% | 22 | 0 |
| *DCC* | 30 | 100.0% | 30 | 0 |
| *EGFR* | 32 | 100.0% | 32 | 0 |
| *ELMO1* | 25 | 93.0% | 24 | 1 |
| *EP300* | 31 | 100.0% | 31 | 0 |
| *EPB41L3* | 25 | 100.0% | 25 | 0 |
| *EPHA2* | 17 | 100.0% | 17 | 0 |
| *ERBB4* | 29 | 100.0% | 29 | 0 |
| *FAT1* | 29 | 100.0% | 29 | 0 |
| *FBXW7* | 14 | 100.0% | 14 | 0 |
| *FLG* | 2 | 100.0% | 2 | 0 |
| *FLNA* | 48 | 100.0% | 48 | 0 |
| *FRG1B* | 8 | 100.0% | 8 | 0 |
| *HLA-A* | 8 | 100.0% | 8 | 0 |
| *HLA-B* | 8 | 100.0% | 8 | 0 |
| *HRAS* | 5 | 100.0% | 5 | 0 |
| *KEAP1* | 5 | 100.0% | 5 | 0 |
| *LAMA1* | 63 | 100.0% | 63 | 0 |
| *LAMA2* | 67 | 99.8% | 66 | 1 |
| *LAMA4* | 44 | 100.0% | 44 | 0 |
| *LEPR* | 21 | 94.7% | 20 | 1 |
| *LRP2* | 79 | 100.0% | 79 | 0 |
| *LRP6* | 23 | 100.0% | 23 | 0 |
| *MMP12* | 10 | 100.0% | 10 | 0 |
| *MROH2B* | 42 | 100.0% | 42 | 0 |
| *MST1L* | 15 | 100.0% | 15 | 0 |
| *NBPF1* | 23 | 100.0% | 23 | 0 |
| *NCOR2* | 50 | 100.0% | 50 | 0 |
| *NFE2L2* | 6 | 96.7% | 5 | 1 |
| *NOTCH1* | 34 | 100.0% | 34 | 0 |
| *NOTCH2* | 38 | 100.0% | 38 | 0 |
| *NOTCH3* | 33 | 100.0% | 33 | 0 |
| *NPAP1* | 1 | 100.0% | 1 | 0 |
| *NSD1* | 25 | 98.2% | 23 | 2 |
| *PARG* | 18 | 100.0% | 18 | 0 |
| *PCDHGB4* | 4 | 100.0% | 4 | 0 |
| *PIK3CA* | 20 | 100.0% | 20 | 0 |
| *PRB2* | 3 | 100.0% | 3 | 0 |
| *PRKDC* | 87 | 100.0% | 87 | 0 |
| *RASA1* | 30 | 98.7% | 29 | 1 |
| *RB1* | 27 | 100.0% | 27 | 0 |
| *ROCK1* | 33 | 100.0% | 33 | 0 |
| *RUNX1T1* | 20 | 98.1% | 19 | 1 |
| *SEC22B* | 5 | 100.0% | 5 | 0 |
| *SEMA5A* | 23 | 100.0% | 23 | 0 |
| *SULF1* | 21 | 100.0% | 21 | 0 |
| *TGFBR2* | 9 | 100.0% | 9 | 0 |
| *TP53* | 14 | 94.3% | 13 | 1 |
| *TRIM51* | 6 | 100.0% | 6 | 0 |
| *ZNF750* | 2 | 100.0% | 2 | 0 |
| *ZNF761* | 3 | 100.0% | 3 | 0 |
| *ZNF845* | 5 | 98.1% | 4 | 1 |

**Supplementary Table S2.** Clinical characteristics of 73 prospective and 103 retrospective OSCC patients. Percentages for groups are shown in brackets. NSND = non-smoker and non-drinker. **p* < 0.05.

| Characteristic |  | Prospective patients  (n = 73) | Retrospective patients (n=103) | *p* |
| --- | --- | --- | --- | --- |
| Gender | Female | 31 (42.5) | 45 (43.7) | 0.879 |
|  | Male | 42 (57.5) | 58 (56.3) |  |
| Age | Median (range) | 67 (34 - 89) | 66 (38 - 99) | 0.472 |
|  | Non-smoker | 40 (54.8) | 46 (44.7) | 0.221 |
|  | Non-drinker | 44 (60.3) | 35 (34) | < 0.001* |
|  | NSND | 32 (43.8) | 27 (26.2) | 0.016* |
| T stage | 1 | 15 (20.5) | 24 (23.3) | 0.107 |
|  | 2 | 23 (31.5) | 43 (41.7) |  |
|  | 3 | 4 (5.5) | 10 (9.7) |  |
|  | 4 | 31 (42.5) | 26 (25.2) |  |
| N stage | N0 | 42 (57.5) | 73 (70.9) | 0.078 |
|  | N+ | 31 (42.5) | 30 (29.1) |  |
| AJCC stage | I | 10 (13.7) | 22 (21.4) | 0.069 |
|  | II | 16 (21.9) | 34 (33.0) |  |
|  | III | 16 (21.9) | 21 (20.4) |  |
|  | IV | 31 (42.5) | 26 (25.2) |  |
| Perineural invasion | Present | 14 (19.2) | 18 (17.5) | 0.844 |
|  | Absent | 59 (80.8) | 85 (82.5) |  |
| Lymphovascular invasion | Present | 6 (8.2) | 12 (11.7) | 0.615 |
|  | Absent | 67 (91.8) | 91 (88.3) |  |
| Extracapsular spread | Present | 9 (12.3) | 11 (10.7) | 0.811 |
|  | Absent | 64 (87.7) | 92 (89.3) |  |
| HPV status | Positive | 0 (0) | 3 (2.9) | 0.268 |
|  | Negative | 73 (100) | 100 (97.1) |  |
| Radiotherapy | Yes | 47 (64.4) | 63 (61.2) | 0.752 |
|  | No | 26 (35.6) | 40 (38.8) |  |
| Chemotherapy | Yes | 14 (19.2) | 25 (24.3) | 0.466 |
|  | No | 59 (80.8) | 78 (75.7) |  |

**Supplementary Table S3.** Clinical characteristics of HPV-negative OSCC patients (n = 173) compared against HPV-positive patients (n = 3). NSND = non-smoker and non-drinker. **p* <0.05.

| Characteristic |  | HPV-negative  (n = 173) | HPV-positive (n = 3) | *p* |
| --- | --- | --- | --- | --- |
| Gender | Female | 75 (43.4) | 1 (33.3) | 1.00 |
|  | Male | 98 (56.6) | 2 (66.7) |  |
| Age | Median (range) | 66 (34 - 99) | 60 (54 - 76) | 0.627 |
|  | Non-smoker | 85 (49.1) | 1 (33.3) | 1.00 |
|  | Non-drinker | 78 (45.1) | 1 (33.3) | 1.00 |
|  | NSND | 58 (33.5) | 1 (33.3) | 1.00 |
| T stage | 1 | 38 (22.0) | 1 (33.3) | 0.581 |
|  | 2 | 64 (37.0) | 2 (66.7) |  |
|  | 3 | 14 (8.1) | 0 (0) |  |
|  | 4 | 57 (32.9) | 0 (0) |  |
| N stage | N0 | 117 (67.6) | 3 (100) | 0.552 |
|  | N+ | 56 (32.4) | 0 (0) |  |
| TNM stage | I | 31 (17.9) | 1 (33.3) | 0.296 |
|  | II | 48 (27.7) | 2 (66.7) |  |
|  | III | 37 (21.4) | 0 (0) |  |
|  | IV | 57 (32.9) | 0 (0) |  |
| Perineural invasion | Present | 31 (17.9) | 1 (33.3) | 0.454 |
|  | Absent | 142 (82.1) | 2 (66.7) |  |
| Lymphovascular invasion | Present | 18 (10.4) | 0 (0) | 1.00 |
|  | Absent | 155 (89.6) | 3 (100) |  |
| Extracapsular spread | Present | 20 (11.6) | 0 (0) | 1.00 |
|  | Absent | 153 (88.4) | 3 (100) |  |
| Radiotherapy | Yes | 108 (62.4) | 2 (66.7) | 1.00 |
|  | No | 65 (37.6) | 1 (33.3) |  |
| Chemotherapy | Yes | 37 (21.4) | 2 (66.7) | 0.124 |
|  | No | 136 (78.6) | 1 (33.3) |  |

**Supplementary Table S4.** Clinical characteristics of OSCC patients in the NSND group (n = 59) compared to the SD group (n = 117). NSND = non-smoker and non-drinker; SD = smokers and/or drinker. **p* <0.05.

| Characteristic |  | SD  (n = 117) | NSND  (n = 59) | *p* |
| --- | --- | --- | --- | --- |
| Gender | Female | 33 (28.2) | 43 (72.9) | <0.001* |
|  | Male | 84 (71.8) | 16 (27.1) |  |
| Age | Median (range) | 65 (38 - 90) | 73 (34 - 99) | 0.004* |
| T stage | 1 | 24 (20.5) | 15 (25.4) | 0.131 |
|  | 2 | 49 (41.9) | 17 (28.8) |  |
|  | 3 | 6 (5.1) | 8 (13.6) |  |
|  | 4 | 38 (32.5) | 19 (32.2) |  |
| N stage | N0 | 76 (65.0) | 39 (66.1) | 1.00 |
|  | N+ | 41 (35.0) | 20 (33.9) |  |
| TNM stage | I | 21 (17.9) | 11 (18.6) | 0.887 |
|  | II | 35 (29.9) | 15 (25.4) |  |
|  | III | 23 (19.7) | 14 (23.7) |  |
|  | IV | 38 (32.5) | 19 (32.2) |  |
| Perineural invasion | Present | 20 (17.1) | 12 (20.3) | 0.680 |
|  | Absent | 97 (82.9) | 47 (79.7) |  |
| Lymphovascular invasion | Present | 11 (9.4) | 7 (11.9) | 0.607 |
|  | Absent | 106 (90.6) | 52 (88.1) |  |
| Extracapsular spread | Present | 13 (11.1) | 7 (11.9) | 1.00 |
|  | Absent | 104 (88.9) | 52 (88.1) |  |
| HPV status | Positive | 2 (1.7) | 1 (1.7) | 1.00 |
|  | Negative | 115 (98.3) | 58 (98.3) |  |
| Radiotherapy | Yes | 72 (61.5) | 38 (64.4) | 0.744 |
|  | No | 45 (38.5) | 21 (35.6) |  |
| Perineural invasion | Present | 28 (23.9) | 11 (18.6) | 0.564 |
|  | Absent | 89 (76.1) | 48 (81.4) |  |

**Supplementary Table S5.** Univariate and multivariate and Cox proportional hazards analyses assessing smoking/drinking status and clinicopathologic variables in 176 OSCC patients. Extracapsular spread was excluded as all patients with ECS were lymph node positive by definition. NSND = non-smoker and non-drinker; SD = smokers and/or drinker; LN = lymph node; PNI = perineural invasion; LVI = lymphovascular invasion; HR = hazard ratio, AHR = adjusted hazard ratio, CI = confidence interval; **p* < 0.05.

| Characteristic | Univariate analysis | | | Multivariate analysis | | |
| --- | --- | --- | --- | --- | --- | --- |
|  | HR | 95% CI | p | AHR | 95% CI | p |
| NSND vs SD | 1.7 | 1.0 - 2.8 | 0.050* | 1.1 | 0.6 - 2.1 | 0.685 |
| Male vs female | 0.8 | 0.5 - 1.3 | 0.406 | 1.1 | 0.6 - 1.9 | 0.839 |
| Age (in decades) | 1.7 | 1.2 - 1.8 | <0.001* | 1.6 | 1.3 - 2.0 | <0.001* |
| T3/4 vs T1/2 | 2.9 | 1.7 - 5.0 | <0.001* | 2.5 | 1.5 - 4.3 | 0.001* |
| LN+ vs LN- | 2.3 | 1.4 - 3.8 | 0.001* | 2.1 | 1.2 - 3.7 | 0.013* |
| PNI+ vs PNI- | 1.7 | 1.0 - 3.1 | 0.064 | 1.5 | 0.8 - 2.8 | 0.187 |
| LVI+ vs LVI- | 2.0 | 1.0 - 4.0 | 0.064 | 1.3 | 0.6 - 3.0 | 0.485 |

**Supplementary Table S6.** Pathogenicity predictions by the PolyPhen-2 algorithm for missense mutations detected in tumours from 176 OSCC patients. Missense mutations with a score > 0.85 were classified as high pathogenicity, and missense mutations with a PolyPhen-2 score < 0.85 were classified as low pathogenicity. Nonsense/indel mutations identified in tumours are shown separately.

|  |  | Missense mutations | | | Nonsense |
| --- | --- | --- | --- | --- | --- |
| Gene | No of mutations | Median PolyPhen-2 score | Low pathogenicity | High pathogenicity | mutations & Indels |
| *TP53* | 106 | 1.00 | 15 (14.2) | 52 (49.1) | 39 (36.8) |
| *CDKN2A* | 42 | 1.00 | 3 (7.1) | 9 (21.4) | 30 (71.4) |
| *FLG* | 39 | 0.22 | 27 (69.2) | 11 (28.2) | 1 (2.6) |
| *NOTCH1* | 30 | 1.00 | 5 (16.7) | 9 (30.0) | 16 (53.3) |
| *FAT1* | 26 | 0.39 | 4 (15.4) | 6 (23.1) | 16 (61.5) |
| *NBPF1* | 21 | 0.29 | 20 (95.2) | 1 (4.8) | 0 (0) |
| *PIK3CA* | 19 | 0.99 | 6 (31.6) | 13 (68.4) | 0 (0) |
| *LRP2* | 15 | 0.95 | 5 (33.3) | 9 (60.0) | 1 (6.7) |
| *CASP8* | 14 | 0.00 | 6 (42.9) | 1 (7.1) | 7 (50.0) |
| *ATP10A* | 13 | 0.65 | 8 (61.5) | 5 (38.5) | 0 (0) |
| *NOTCH2* | 13 | 1.00 | 4 (30.8) | 7 (53.8) | 2 (15.4) |
| *BRCA2* | 12 | 0.20 | 7 (58.3) | 2 (16.7) | 3 (25.0) |
| *EP300* | 12 | 1.00 | 1 (8.3) | 9 (75.0) | 2 (16.7) |
| *NCOR2* | 12 | 1.00 | 5 (41.7) | 5 (41.7) | 2 (16.7) |
| *PRKDC* | 12 | 0.95 | 9 (75.0) | 3 (25.0) | 0 (0) |
| *EPB41L3* | 11 | 0.98 | 4 (36.4) | 7 (63.6) | 0 (0) |
| *FLNA* | 11 | 0.99 | 4 (36.4) | 7 (63.6) | 0 (0) |
| *LAMA1* | 11 | 0.55 | 6 (54.5) | 4 (36.4) | 1 (9.1) |
| *MST1L* | 11 | 0.01 | 5 (45.5) | 1 (9.1) | 5 (45.5) |
| *EPHA2* | 9 | 1.00 | 2 (22.2) | 3 (33.3) | 4 (44.4) |
| *LAMA2* | 9 | 0.97 | 2 (22.2) | 6 (66.7) | 1 (11.1) |
| *LAMA4* | 9 | 0.14 | 4 (44.4) | 4 (44.4) | 1 (11.1) |
| *NSD1* | 9 | 0.68 | 0 (0) | 5 (55.6) | 4 (44.4) |

**Supplementary Table S7.** Gene amplifications and deletions in 176 OSCC patients as determined by ExomeDepth. * indicates where copy number changes are exclusively amplifications or deletions and at least 5 were found; ^indicate CNVs found in the prospective cohort but not in the retrospective cohort.

| Gene | Deletions | Amplifications |  |
| --- | --- | --- | --- |
|  | (n=176) | |  |
| *AJUBA* | 1 (0.6) | 0 (0) |  |
| *ATP10A* | 3 (1.7) | 0 (0) |  |
| *ATR* | 3 (1.7) | 3 (1.7) |  |
| *BRCA2* | 9 (5.1) | 0 (0) | * |
| *CACNA1C* | 1 (0.6) | 1 (0.6) |  |
| *CACNA1D* | 3 (1.7) | 0 (0) |  |
| *CACNA1E* | 0 (0) | 3 (1.7) |  |
| *CACNA2D1* | 13 (7.4) | 0 (0) | ^* |
| *CDKN2A* | 7 (4.0) | 0 (0) | * |
| *DCC* | 1 (0.6) | 0 (0) |  |
| *EGFR* | 0 (0) | 16 (9.1) | * |
| *ELMO1* | 0 (0) | 1 (0.6) |  |
| *EPB41L3* | 0 (0) | 4 (2.3) |  |
| *EPHA2* | 3 (1.7) | 0 (0) |  |
| *ERBB4* | 1 (0.6) | 0 (0) |  |
| *FAT1* | 2 (1.1) | 0 (0) |  |
| *FBXW7* | 18 (10.2) | 3 (1.7) | ^ |
| *FLNA* | 1 (0.6) | 4 (2.3) |  |
| *HRAS* | 1 (0.6) | 0 (0) |  |
| *LAMA1* | 2 (1.1) | 4 (2.3) |  |
| *LEPR* | 1 (0.6) | 0 (0) |  |
| *LRP2* | 0 (0) | 1 (0.6) |  |
| *LRP6* | 0 (0) | 1 (0.6) |  |
| *MMP12* | 0 (0) | 10 (5.7) | * |
| *NCOR2* | 5 (2.8) | 14 (8.0) |  |
| *NFE2L2* | 0 (0) | 4 (2.3) |  |
| *NOTCH1* | 5 (2.8) | 11 (6.3) |  |
| *NOTCH2* | 0 (0) | 2 (1.1) |  |
| *NOTCH3* | 7 (4.0) | 3 (1.7) |  |
| *NSD1* | 2 (1.1) | 0 (0) |  |
| *PIK3CA* | 0 (0) | 2 (1.1) |  |
| *PRKDC* | 0 (0) | 8 (4.5) | * |
| *RASA1* | 23 (13.1) | 1 (0.6) | ^ |
| *RB1* | 23 (13.1) | 1 (0.6) | ^ |
| *ROCK1* | 5 (2.8) | 0 (0) |  |
| *RUNX1T1* | 0 (0) | 7 (4.0) |  |
| *SEMA5A* | 0 (0) | 2 (1.1) |  |
| *SULF1* | 0 (0) | 1 (0.6) |  |
| *TGFBR2* | 1 (0.6) | 1 (0.6) |  |
| *TP53* | 1 (0.6) | 1 (0.6) |  |

**Supplementary Table S8.** Univariate and multivariate and Cox proportional hazards analyses assessing genes mutated in at least 5% (9/176) of OSCC patients. Multivariate models are adjusted for clinicopathologic variables. HR = hazard ratio, AHR = adjusted hazard ratio, CI = confidence interval; * *p* < 0.05.

| Gene |  | Univariate analysis | | | | Multivariate analysis | | | |
| --- | --- | --- | --- | --- | --- | --- | --- | --- | --- |
|  | n | HR | 95% CI | p |  | AHR | 95% CI | p |  |
| *ATP10A* | 13 | 0.6 | 0.2 - 1.9 | 0.370 |  | 0.7 | 0.2 - 2.2 | 0.497 |  |
| *BRCA2* | 12 | 0.7 | 0.2 - 2.1 | 0.480 |  | 1.2 | 0.4 - 3.9 | 0.805 |  |
| *CASP8* | 14 | 1.7 | 0.8 - 3.8 | 0.175 |  | 1.7 | 0.7 - 3.8 | 0.216 |  |
| *CDKN2A* | 42 | 1.5 | 0.8 - 2.6 | 0.168 |  | 0.9 | 0.5 - 1.5 | 0.611 |  |
| *EP300* | 12 | 0.7 | 0.2 - 2.2 | 0.523 |  | 0.7 | 0.2 - 2.2 | 0.509 |  |
| *EPB41L3* | 11 | 0.7 | 0.2 - 2.2 | 0.521 |  | 0.8 | 0.2 - 2.7 | 0.725 |  |
| *EPHA2* | 9 | 1.5 | 0.6 - 4.2 | 0.413 |  | 1.2 | 0.4 - 3.4 | 0.694 |  |
| *FAT1* | 26 | 1.3 | 0.6 - 2.5 | 0.524 |  | 1.4 | 0.7 - 2.8 | 0.406 |  |
| *FLG* | 39 | 1.2 | 0.7 - 2.1 | 0.608 |  | 1.4 | 0.8 - 2.6 | 0.268 |  |
| *FLNA* | 11 | 0.7 | 0.2 - 2.1 | 0.497 |  | 0.5 | 0.1 - 1.5 | 0.198 |  |
| *LAMA1* | 11 | 1.0 | 0.4 - 2.9 | 0.942 |  | 1.1 | 0.4 - 3.3 | 0.804 |  |
| *LAMA2* | 9 | 2.1 | 0.8 - 5.3 | 0.109 |  | 2.6 | 1.0 - 7.2 | 0.057 |  |
| *LAMA4* | 9 | 1.1 | 0.3 - 3.4 | 0.932 |  | 1.9 | 0.6 - 6.5 | 0.302 |  |
| *LRP2* | 15 | 0.9 | 0.4 - 2.3 | 0.856 |  | 0.8 | 0.3 - 2.0 | 0.580 |  |
| *MST1L* | 11 | 1.0 | 0.4 - 2.7 | 0.944 |  | 1.3 | 0.5 - 3.6 | 0.643 |  |
| *NBPF1* | 21 | 0.6 | 0.2 - 1.5 | 0.300 |  | 0.7 | 0.3 - 1.9 | 0.507 |  |
| *NCOR2* | 12 | 0.7 | 0.2 - 2.2 | 0.524 |  | 0.4 | 0.1 - 1.4 | 0.158 |  |
| *NOTCH1* | 30 | 1.1 | 0.6 - 2.2 | 0.706 |  | 1.0 | 0.5 - 2.0 | 0.973 |  |
| *NOTCH2* | 13 | 0.6 | 0.2 - 1.8 | 0.344 |  | 0.6 | 0.2 - 1.9 | 0.377 |  |
| *NSD1* | 9 | 1.3 | 0.5 - 3.5 | 0.635 |  | 1.2 | 0.4 - 3.4 | 0.754 |  |
| *PIK3CA* | 19 | 2.0 | 1.0 - 3.9 | 0.050* |  | 1.4 | 0.7 - 2.9 | 0.303 |  |
| *PRKDC* | 12 | 0.2 | 0.0 - 1.3 | 0.090 |  | 0.2 | 0.0 - 1.2 | 0.069 |  |
| *TP53* | 106 | 1.4 | 0.8 - 2.3 | 0.244 |  | 1.1 | 0.6 - 2.0 | 0.708 |  |


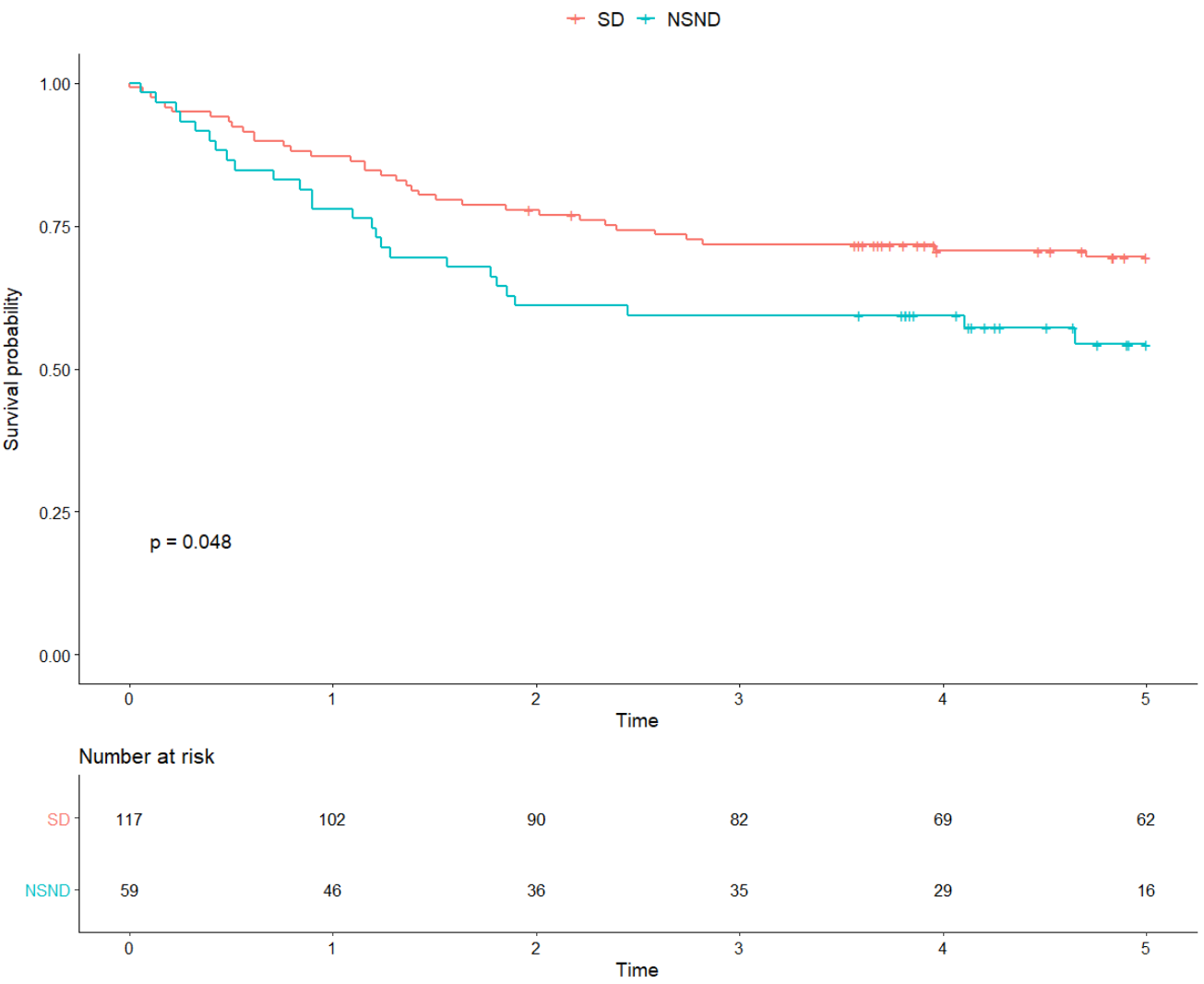


**Supplementary Figure S1.** Kaplan-Meier survival curves for 176 OSCC patients by smoking/drinking status. NSND = non-smoker and non-drinker; SD = smokers and/or drinker. P value is for the log rank test.


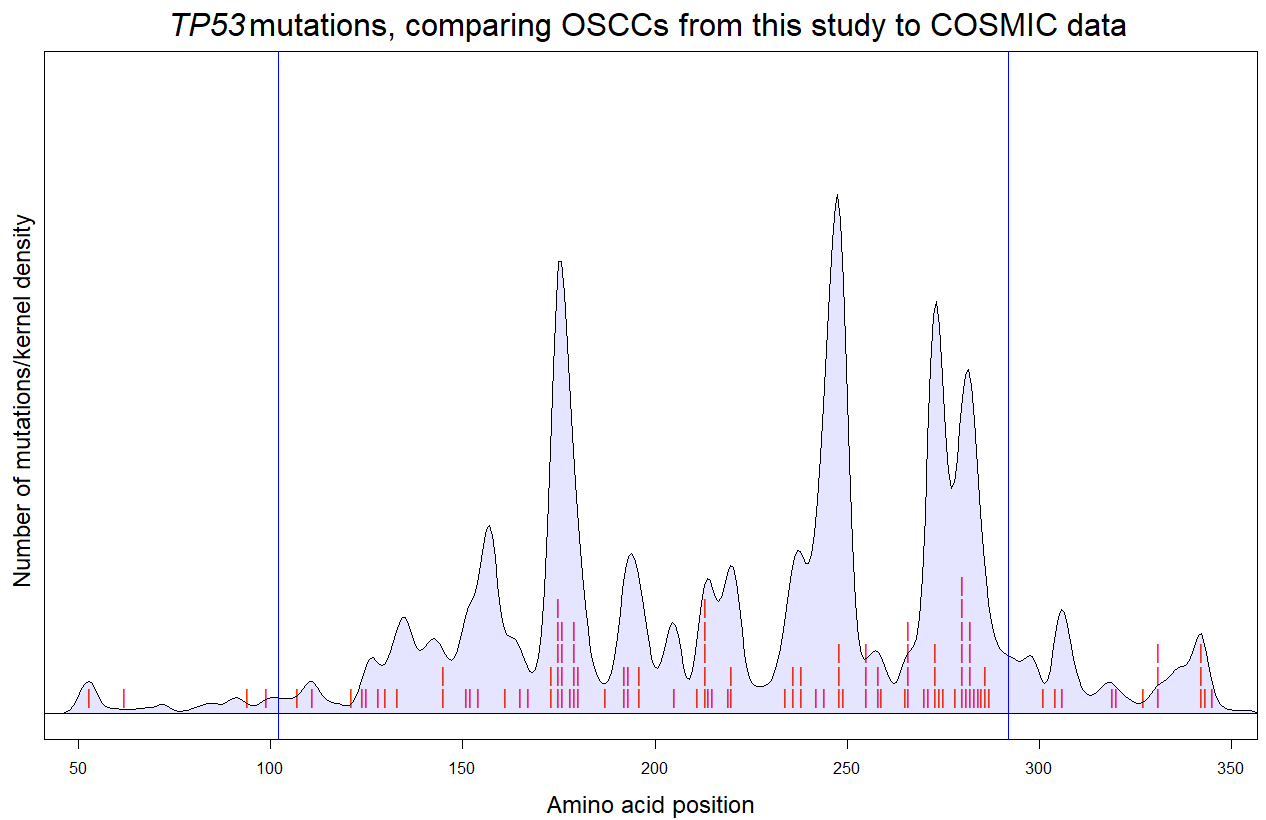


**Supplementary Figure S2.** Amino acid positions for *TP53* mutations (n=118) detected in our OSCC cohort against mutations reported for aerodigestive tumours in the COSMIC database (n = 2914). The red bars indicate mutations in our OSCC cohort, the density plot represents abundance of mutations in the COSMIC data. The two blue lines indicate the region of the DNA binding domain.


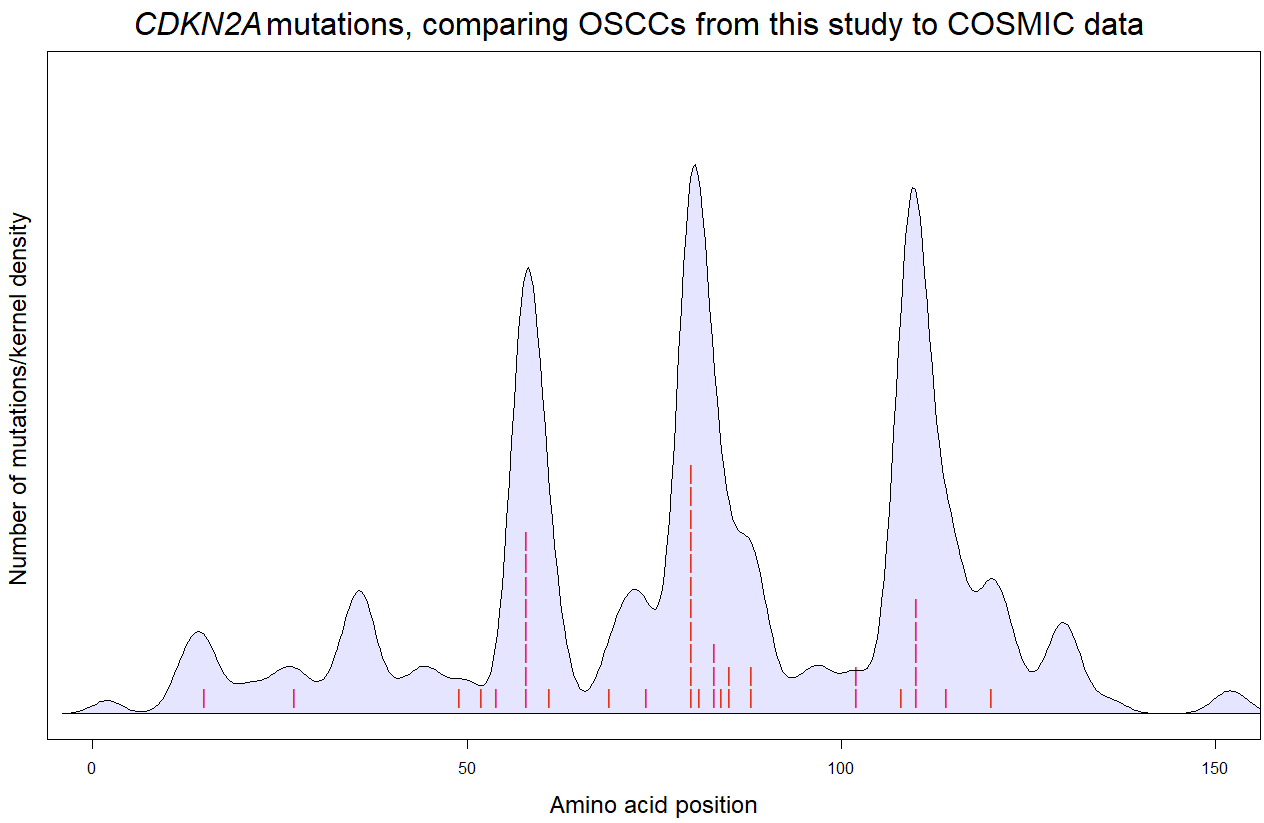


**Supplementary Figure S3.** Amino acid positions for *CDKN2A* mutations (n=46) detected in our OSCC cohort against mutations reported for aerodigestive tumours in the COSMIC database (n = 511). The red bars indicate mutations in our OSCC cohort, the density plot represents abundance of mutations in the COSMIC data.


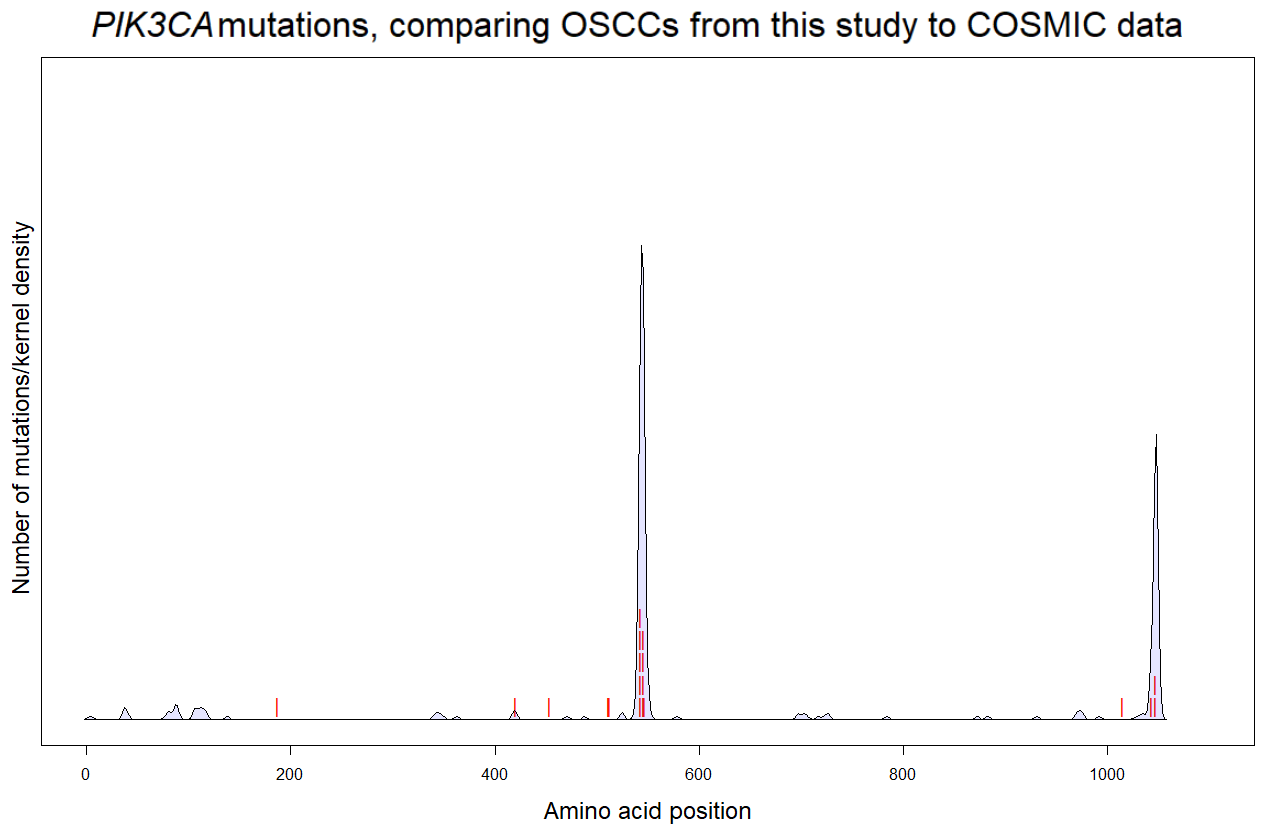


**Supplementary Figure S4.** Amino acid positions for *PIK3CA* mutations (n=19) detected in our OSCC cohort against mutations reported for aerodigestive tumours in the COSMIC database (n = 341). The red bars indicate mutations in our OSCC cohort, the density plot represents abundance of mutations in the COSMIC data.


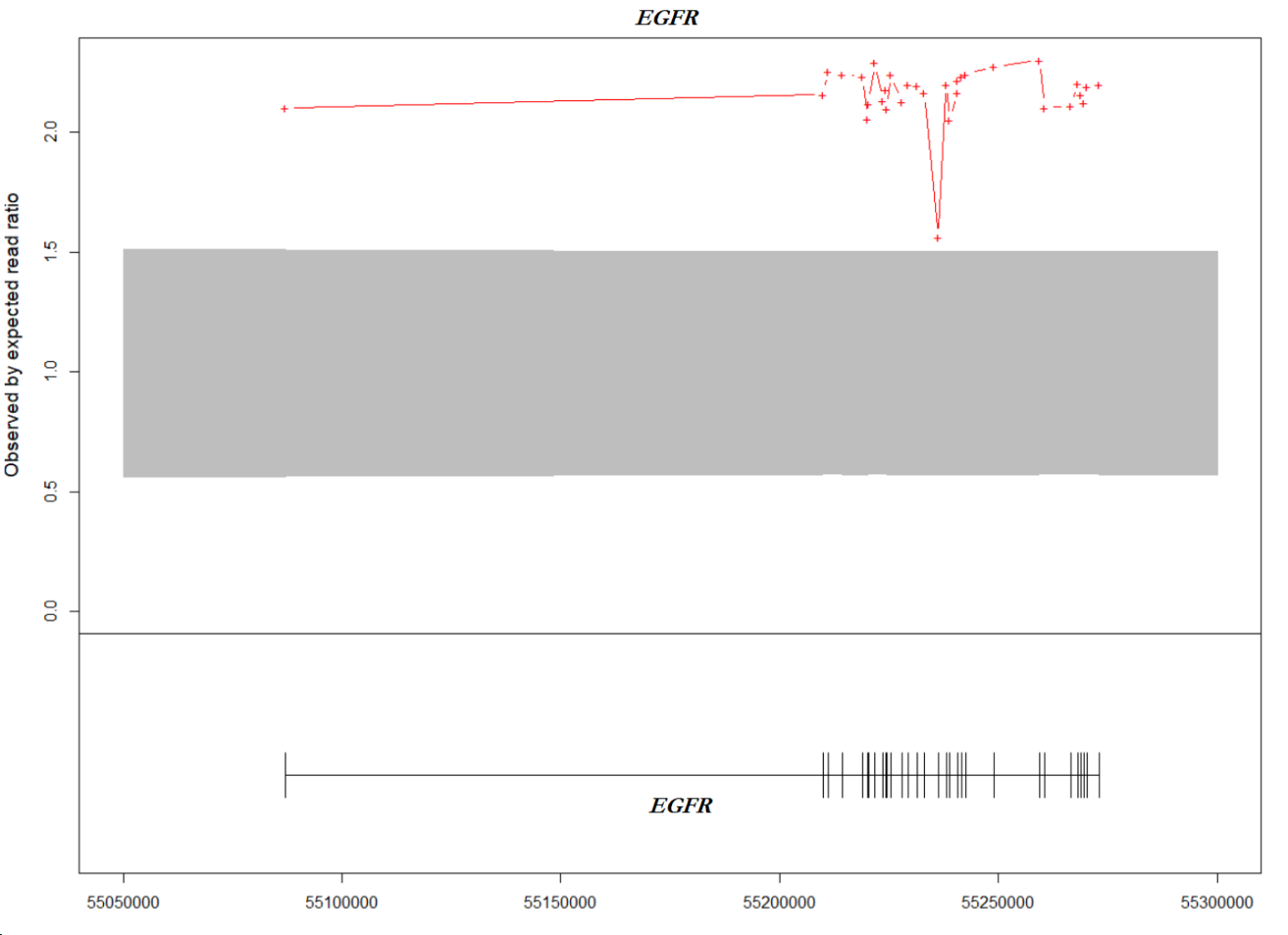


**Supplementary Figure S5.** ExomeDepth CNV plot for a representative OSCC sample with a detected *EGFR* amplification. Observed over expected read ratio is plotted on the Y-axis. Reads in this sample across the gene are denoted by the red line, with the grey zone indicating the 95% confidence interval within which gene amplification is unlikely.


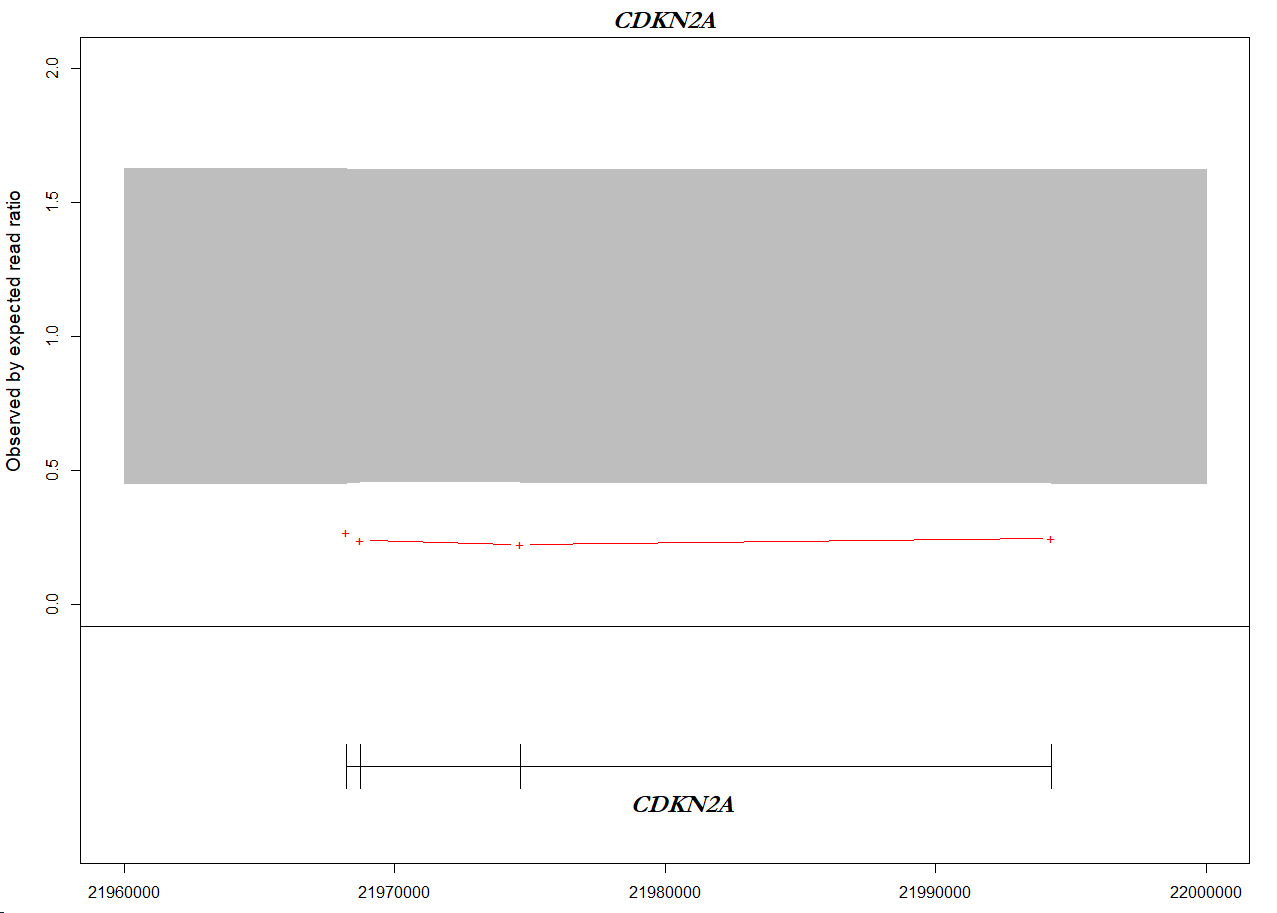


**Supplementary Figure S6.** ExomeDepth CNV plot for a representative OSCC sample with a detected *CDKN2A* deletion. Observed over expected read ratio is plotted on the Y-axis. Reads in this sample across the gene are denoted by the red line, with the grey zone indicating the 95% confidence interval within which gene amplification is unlikely.


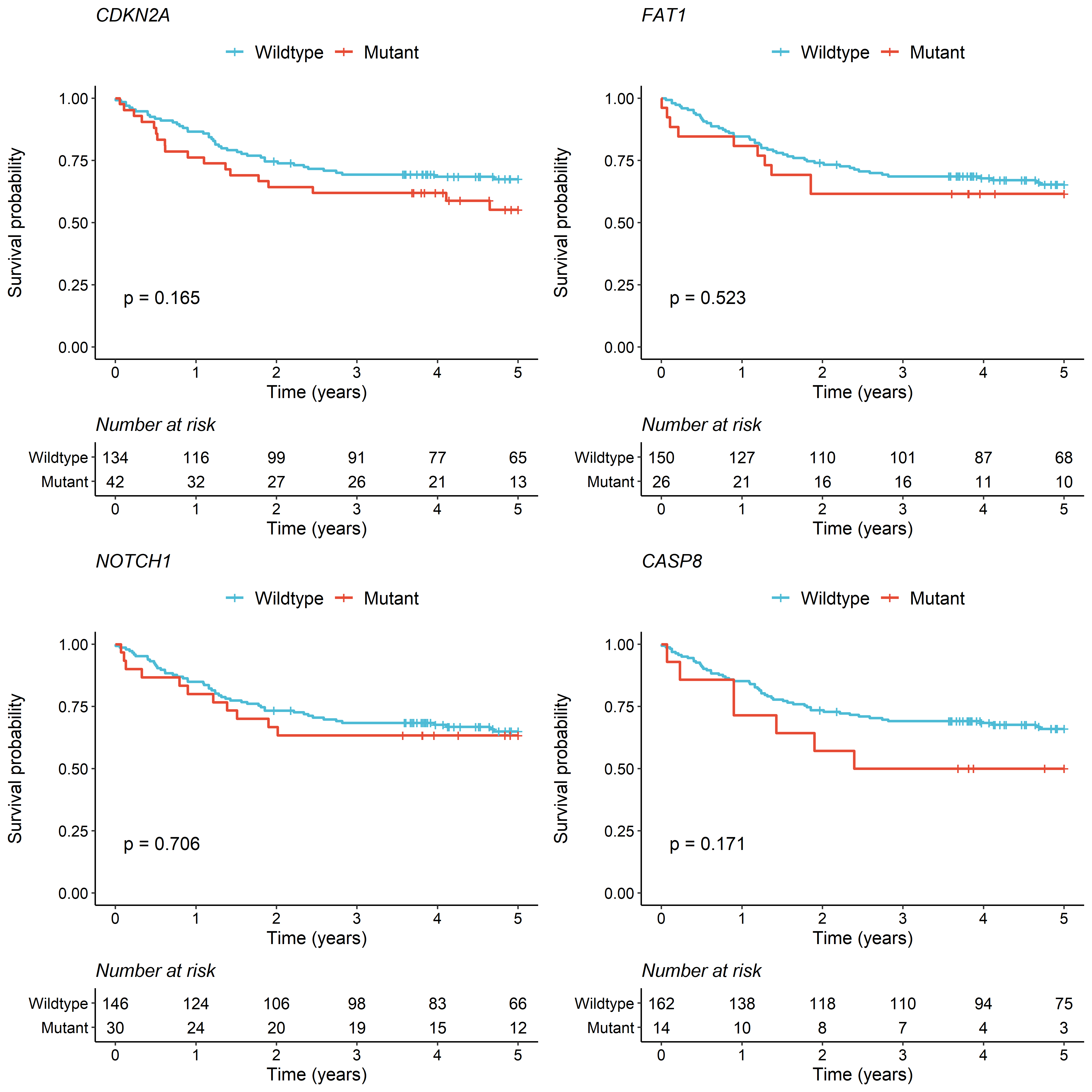


**Supplementary Figure S7.** Kaplan-Meier survival curves for 176 OSCC patients by *CDKN2A*, *FAT1*, *NOTCH1* or *CASP8* mutation status. P values are for the log rank test.


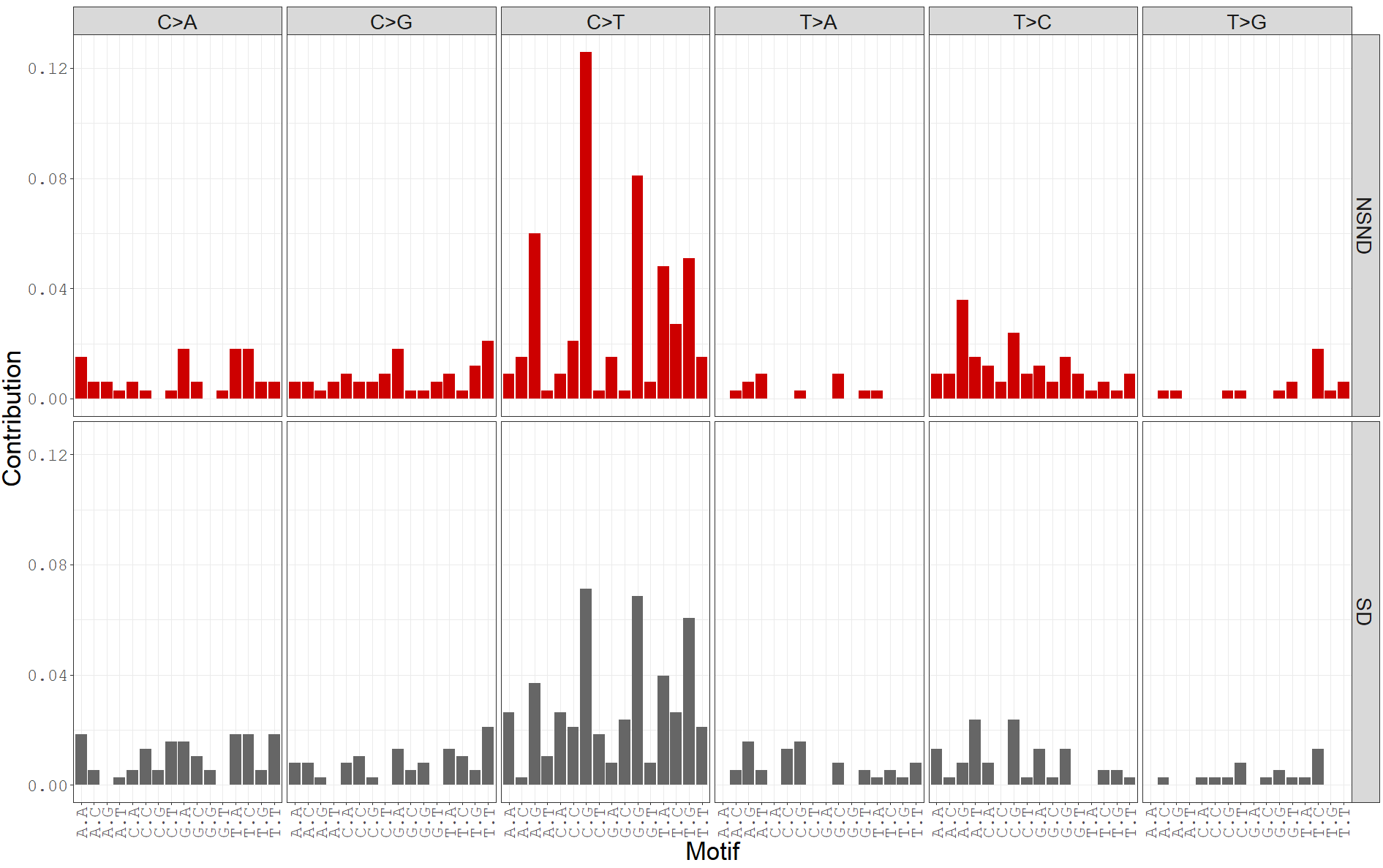


**Supplementary Figure S8.** Spectrum of missense mutations in OSCC tumours from NSND patients (n=59) (red, top panel) as compared to SD (n=117) patients (grey, bottom panel). NSND = non-smoker and non-drinker; SD = smokers and/or drinker.
